# Bakua: Tinea Imbricata in the Solomon Islands

**DOI:** 10.4269/ajtmh.14-0770

**Published:** 2015-05-06

**Authors:** Daniel Mason, Michael Marks

**Affiliations:** Royal Children's Hospital, Melbourne, Centre for International Child Health, Parkville, Victoria, Australia; Clinical Research Department, Faculty of Infectious and Tropical Diseases, London School of Hygiene and Tropical Medicine, London, United Kingdom; The Hospital for Tropical Diseases, London, United Kingdom

**Figure 1.**
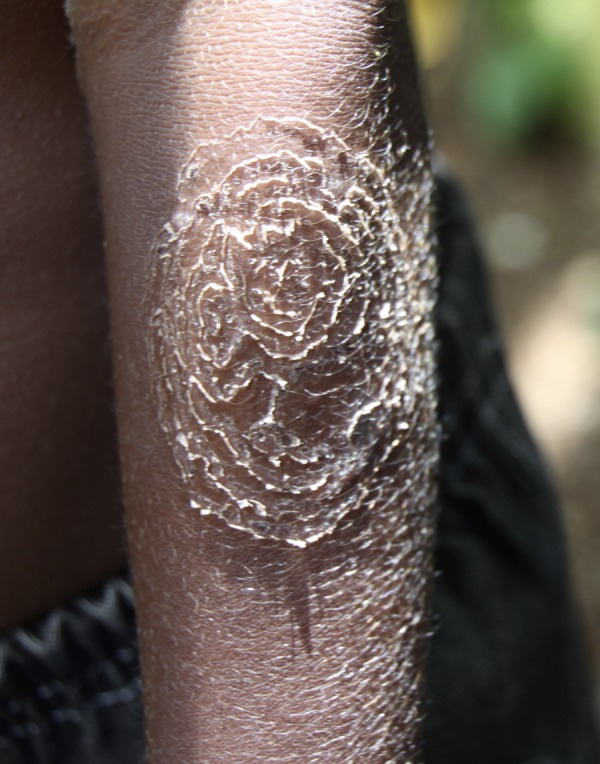
Classical lesion of tinea imbricata on the arm of a child from the Solomon Islands.

**Figure 2.**
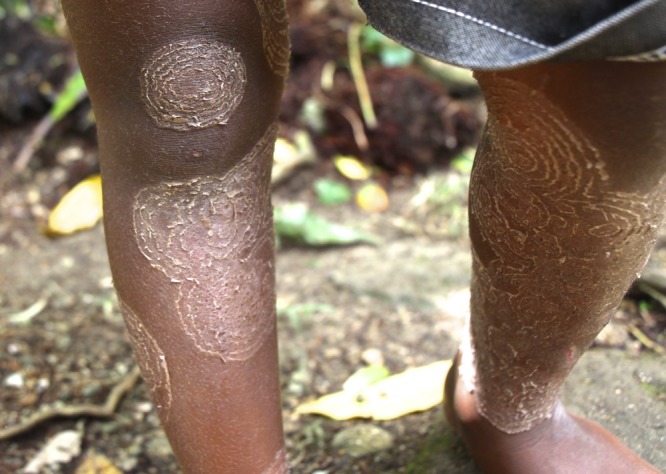
Multiple overlapping lesions of tinea imbricata on the legs of the same child from the western province of the Solomon Islands.

Tinea imbricata or tokelau is a chronic superficial mycosis caused by *Trichophyton concentricum*.^1^ The disease is endemic in the Pacific, including on the Solomon Islands, where it is known as bakua. The disease predominantly affects individuals living in poor rural communities with limited access to hygiene. It is estimated that between 10% and 20% of individuals in some Pacific countries are affected. Tinea imbricata begins in childhood and affects both sexes. Skin lesions can affect the whole body, but the limbs and torso are most commonly involved. Lesions begin as concentric, annular plaques with or without erythema (Figure 1

). Over time, multiple overlapping lesions develop (Figure 2

). Pruritus is frequent. The florid nature of tinea imbricata can make infection socially stigmatizing. The disease has a classical appearance, and diagnosis is clinical, although fungal scrapings and culture are possible. There is thought to be a genetic pre-disposition, with both autosomal-recessive and -dominant inheritance described. Individuals who develop tinea imbricata have impaired immune responses to fungal antigens, but the precise mechanism is not well understood.^1^ Current treatment options are limited, with griseofulvin or oral terbinafine preferred.^2^ Even with prolonged treatment, recurrence rates are high, and a more efficacious treatment regimen is needed.
